# Accuracy and Screw Insertion Time of Robotic-Assisted Cortical Bone Trajectory Screw Placement for Posterior Lumbar Interbody Fusion: A Comparison of Early, Middle, and Late Phases

**DOI:** 10.7759/cureus.32574

**Published:** 2022-12-15

**Authors:** Jun Ueno, Tsutomu Akazawa, Yoshiaki Torii, Tasuku Umehara, Masahiro Iinuma, Atsuhiro Yoshida, Ken Tomochika, Hisateru Niki

**Affiliations:** 1 Department of Orthopaedic Surgery, St. Marianna University School of Medicine, Kawasaki, JPN; 2 Department of Orthopaedic Surgery and Spine Center, St. Marianna University School of Medicine, Kawasaki, JPN

**Keywords:** robotic spine surgery, posterior lumbar fusion, screw insertion time, deviation rate, accuracy rate, cortical bone trajectory, robotic-assisted pedicle screw placement

## Abstract

Introduction

The purpose of this study was to evaluate robotic-assisted cortical bone trajectory (CBT) screw placement. Early, middle, and late phases of robotic-assisted CBT screw placement were compared for accuracy and screw insertion time by comparing time and accuracy in every phase.

Methods

A retrospective review was conducted on the initial 40 patients who underwent spinal fusion using CBT screws in one institution from September 2021 to September 2022 utilizing a spine surgery robot system (Mazor X Stealth Edition, Medtronic Inc., Dublin, Ireland). The inclusion criterion was one- or two-level posterior lumbar interbody fusion (PLIF). Exclusion criteria were 1) patients who underwent posterior-lateral fusion in other segments, 2) patients who underwent additional decompression in other segments, 3) patients who underwent reoperation, and 4) patients with spondylolysis. The deviation of the CBT screw was evaluated on computed tomography (CT) one week after surgery using the Gertzbein-Robbins grade system. The rate of Grade A was considered the perfect accuracy rate, and the rate of penetration of 2 mm or more (Grades C, D, and E) was calculated as the deviation rate. To assess the learning curve, patients were divided into three groups. The first 10 cases were in the early phase group, the subsequent 10 cases were in the middle phase group, and the last 10 cases were in the late phase group. We compared the perfect accuracy rate, deviation rate, operative time, operative time per segment, intraoperative blood loss, registration time, and screw insertion time among the three groups.

Results

Thirty patients met the criteria. Overall, the perfect accuracy (Grade A) rate of the screw was 95.3% and the deviation rate was 1.4%. The perfect accuracy rate was 90.4% in the early phase, 95.5% in the middle phase, and 100% in the late phase. The deviation rate was 3.8% in the early phase, 0% in the middle phase, and 0% in the late phase, and there was no statistically significant difference between the three groups. Among the three groups, the operative time, the operative time per segment, the intraoperative blood loss, and the registration time were not significantly different. There was no significant difference in the screw insertion time among the three groups, but it decreased with experience (early phase: 156.9 ± 54.7 sec, middle phase: 139.9 ± 41.6 sec, and late phase: 106.4 ± 39.9 sec, p=0.060). The screw insertion time of the late phase tended to be shorter than that of the early phase (p=0.052).

Conclusions

The deviation rate of robotic-assisted CBT screw placement with one- or two-level PLIF was 1.4%, which was highly accurate. The deviation rate was 3.8% in the early phase, 0% in the middle phase, and 0% in the late phase. Although the deviation rate was low even in the early period, the screw insertion time in the early 10 cases tended to be longer than that in the late 10 cases. After passing the experience of 10 cases, this study concluded that robotic-assisted CBT screw placement was proficient.

## Introduction

Cortical bone trajectory (CBT) is a novel technique for placing pedicle screws (PSs). The CBT screw passes through the medial to lateral and caudal to cranial passages through the pedicle [1]. Advantages associated with the use of CBT screws were reported to include fewer skin and paraspinal muscle incisions, higher pull-out strength, and lower risk of injury to the superior adjacent facet joints [2].

The CBT screw is inserted toward the superior endplate with 25° to 30° cranial trajectory and 10° lateral trajectory. [3]. Due to the difference from the traditional PS trajectory, computer-assisted devices such as navigation [4-7] and three-dimensional (3D)-printed templates [8-11] are being used. Furthermore, recently, robotic-assisted spine surgery has been performed, which is expected to contribute to improving the safety of the CBT screw [12-16].

The purpose of this study was to clarify the accuracy and screw insertion time of robotic-assisted CBT screw placement. Early, middle, and late phases of robotic-assisted CBT screw placement were compared for accuracy and screw insertion time to better understand the learning curve following the introduction of robotics.

## Materials and methods

Subjects

This study was approved by the institutional review board of St. Marianna University School of Medicine. A retrospective review was conducted on the initial 40 patients who underwent spinal fusion using CBT screws in one institution (St. Marianna University Hospital, Kawasaki, Japan) from September 2021 to September 2022 utilizing a spine surgery robot system (Mazor X Stealth Edition, Medtronic Inc., Dublin, Ireland). The inclusion criterion was one- or two-level posterior lumbar interbody fusion (PLIF). Exclusion criteria were 1) patients who underwent posterior-lateral fusion in other segments, 2) patients who underwent additional decompression in other segments, 3) patients who underwent reoperation, and 4) patients with spondylolysis. We indicated PLIF for lumbar spinal stenosis with instability. The spondylolysis was not indicated because of a separation of pars at the insertion point of the CBT screw.

Surgical workflow

**Figure 1:**
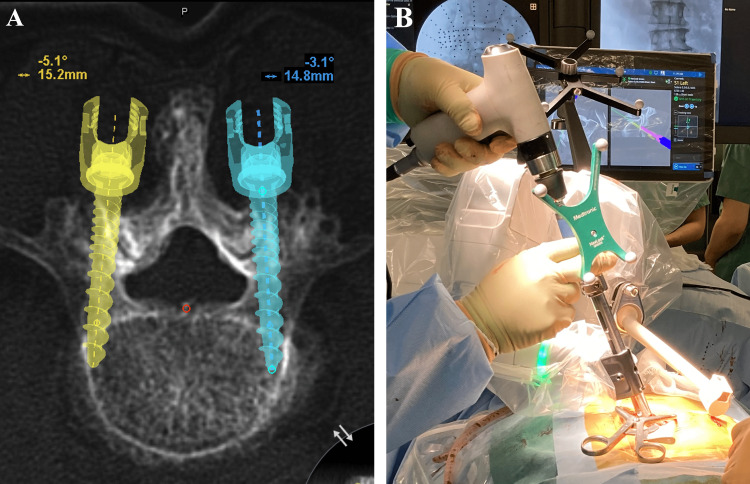
Surgical workflow A: Preoperative CT images were used to plan screw placements using a dedicated application. B: The CBT screw placement was performed without Kirschner wires under the robot arm guidance. CT: Computed tomography; CBT: cortical bone trajectory

Preoperative computed tomography (CT) images were used to plan screw placements using a dedicated application. Surgery was performed in the prone position, and a fusion area was approached through a midline skin incision. The robot arm unit was attached to the bone mount with a Schanz screw (Mazor Robotics Ltd., Israel) on the posterior iliac crest, and a robot reference frame was employed. Preoperative CT image data and intraoperative C-arm images were matched by CT-to-fluoro registration. Drilling, tapping, and screw placement were performed without Kirschner wires under the robot arm guidance (Figure 1). Bone, ligamentum flavum, and disc resections were performed in the segment(s) where PLIF was to be performed, and cage(s) were installed. Two rods were then used to fix the screws. The wound was closed in a normal fashion.

Evaluation

**Figure 2:**
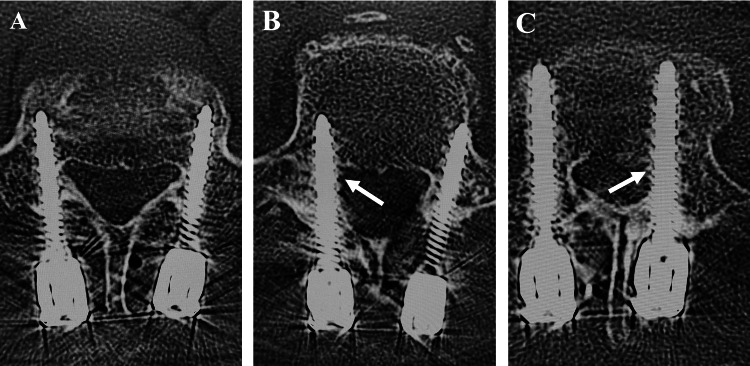
CT evaluation The deviation of the CBT screw was evaluated on CT one week after surgery using the Gertzbein-Robbins grade system [17]. A: Grade A: no breach of the cortical layer of the pedicle. B: Grade B: breaches less than 2 mm. C: Grade C: breaches of 2 mm or more but less than 4 mm. CT: Computed tomography; CBT: cortical bone trajectory

The deviation of the CBT screw was evaluated on CT one week after surgery using the Gertzbein-Robbins grade system: Grade A: no breach of the cortical layer of the pedicle; Grade B: breaches less than 2 mm; Grade C: breaches of 2 mm or more but less than 4 mm; Grade D: breaches of 4 mm or more but less than 6 mm; Grade E: breaches of 6 mm or more (Figure 2) [17]. PS placement was assessed by one author (J.U.) of this study who was blinded to the clinical symptoms. The rate of Grade A was considered the perfect accuracy rate, and the rate of penetration of 2 mm or more (Grades C, D, and E) was calculated as the deviation rate. 

The operative time, operative time per segment, intraoperative blood loss, intraoperative blood loss per segment, registration time, and screw insertion time were evaluated. The registration time was defined as the time from setting up the bone-mounted platform to completing registration. The insertion time was recorded for each CBT screw. The insertion time was defined as the time from making a pilot hole to completing CBT screw placement. In each case, the mean insertion time was defined as the screw insertion time.

Comparison among the early, middle, and late phases

To assess the learning curve, patients were divided into three groups. The first 10 cases were in the early phase group, the subsequent 10 cases were in the middle phase group, and the last 10 cases were in the late phase group. We compared the perfect accuracy rate, deviation rate, operative time, operative time per segment, intraoperative blood loss, registration time, and screw insertion time among the three groups.

Statistical analysis

Continuous variables were expressed as the mean ± standard deviation. A one-way analysis of variance with the post hoc Tukey test was used to compare the three groups. Differences between groups for categorical variables were assessed using the chi-square test. Significant differences were defined as p-value < 0.05. 

## Results

Patient and surgical characteristics

**Figure 3:**
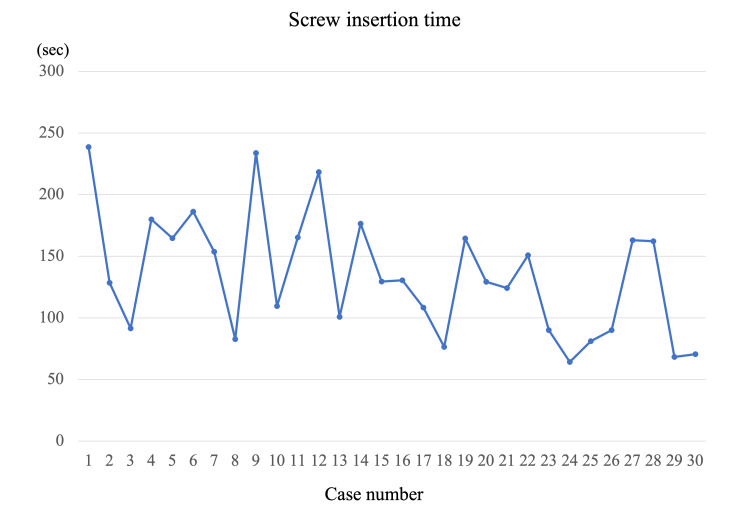
Screw insertion time The screw insertion time declined as the number of surgical cases increased. In each case, the mean insertion time per single screw was defined as the screw insertion time.

Thirty patients met the criteria. The study population consisted of 13 males and 17 females with the mean age at surgery of 65.6 ± 15.4 years (range, 31-87 years). The preoperative diagnoses were lumbar spinal stenosis in 24 patients and lumbar disc herniation in 6 patients. One-level PLIF was performed in 17 patients, and 13 patients underwent two-level PLIF. The mean operative time was 198.7 ± 46.9 min (one-level: 184.1 ± 49.7 min and two-level: 217.9 ± 36.5 min). The intraoperative blood loss was 138.8 ± 120.3 ml (one-level: 93.5 ± 70.2 ml and two-level: 198.0 ± 147.3 ml). The mean screw insertion time was 134.4 ± 49.1 sec. There were no patients who had a pedicle crack during the screw insertion or who required re-insertion of screws after the first placement. The screw insertion time declined as the number of surgical cases increased (Figure 3).

 

There were no significant differences in age at surgery, gender, body mass index, preoperative diagnosis, and the number of fused segments among the three groups (Table 1).

**Table 1:** Patient and surgical characteristics. Normally distributed continuous variables were expressed as the mean ± standard deviation.

	Early phase	Middle phase	Late phase	p
Age at surgery (years)	70.2 ± 11.0	60.0 ± 16.0	66.7± 18.2	0.333
Gender (male/female)	4/6	5/5	4/6	1.000
Body mass index	24.4 ± 4.8	24.3 ± 4.0	24.4 ± 3.9	0.998
Preoperative diagnosis				0.847
Lumbar spinal stenosis	9 patients	7 patients	8 patients	
Lumbar disc herniation	1 patient	3 patients	2 patients	
Number of fused segments				0.266
One-level	5 patients	8 patients	4 patients	
Two-level	5 patients	2 patients	6 patients	
Operative level				
L2-3	1	1	0	
L3-4	7	1	1	
L4-5	8	5	7	
L5-S1	0	5	8	

CBT screw accuracy

Overall, the perfect accuracy rate of the screw was 95.3% and the deviation rate was 1.4%. Table 2 showed the Gertzbein-Robbins grades of the three groups. The perfect accuracy rate was 90.4% in the early phase, 95.5% in the middle phase, and 100% in the late phase. Although there was no statistically significant difference (p=0.059), the perfect accuracy rate increased with experience. The deviation rate was 3.8% in the early phase, 0% in the middle phase, and 0% in the late phase, and there was no statistically significant difference between the three groups. 

**Table 2:** Gertzbein-Robbins grade distribution and screw accuracy.

	Early phase	Middle phase	Late phase	p
Number of screws	52	44	52	
Gertzbein-Robbins grade				
A	47	42	52	
B	3	2	0	
C	2	0	0	
D	0	0	0	
E	0	0	0	
Perfect accuracy rate	90.4%	95.5%	100%	0.059
Deviation rate	3.8%	0%	0%	0.331

Operative time and intraoperative blood loss

**Figure 4:**
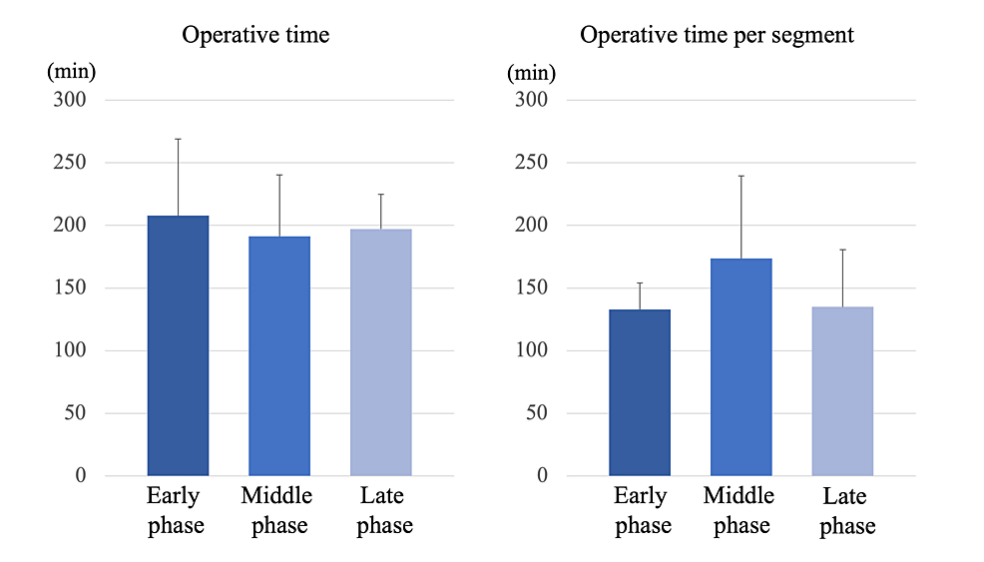
Operative time and operative time per segment Among the three groups, the operative time and the operative time per segment were not significantly different.

Among the three groups, the operative time (early phase: 207.8 ± 61.3 min, middle phase: 191.2 ± 49.0 min, late phase: 197.1 ± 27.7 min, p=0.739) and the operative time per segment (early phase: 132.9 ± 21.2 min, middle phase: 173.6 ± 65.9 min, late phase: 134.9 ± 45.6 min, p=0.120) were not significantly different (Figure 4).

**Figure 5:**
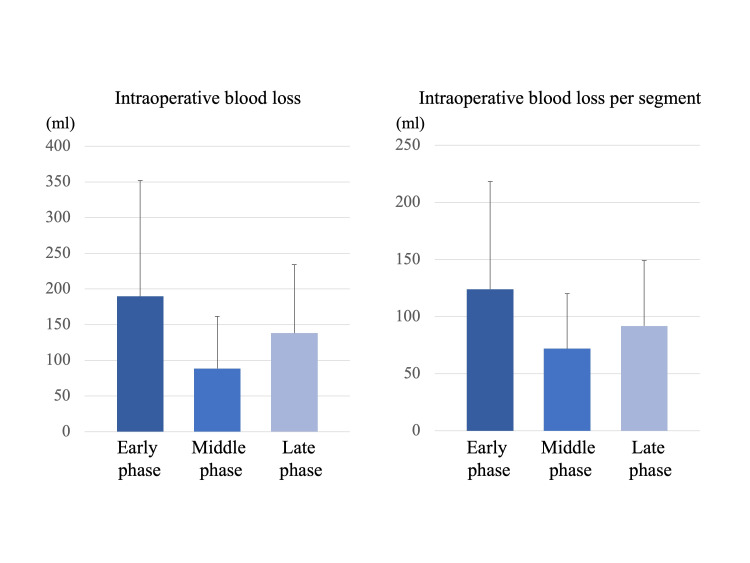
Intraoperative blood loss and intraoperative blood loss per segment There was no significant difference in the intraoperative blood loss and intraoperative blood loss per segment among the three groups.

There was no significant difference in the intraoperative blood loss (early phase: 189.7 ± 162.3 ml, middle phase: 88.5 ± 73.0 ml, late phase: 138.2 ± 96.1 ml, p=0.172) among the three groups (Figure 5). There was also no significant difference in the intraoperative blood loss per segment (early phase: 124.0 ± 94.3 ml, middle phase: 72.0 ± 48.1 ml, late phase: 91.8 ± 57.3 ml, p=0.258) among the three groups (Figure 5).

Registration time and screw insertion time

**Figure 6:**
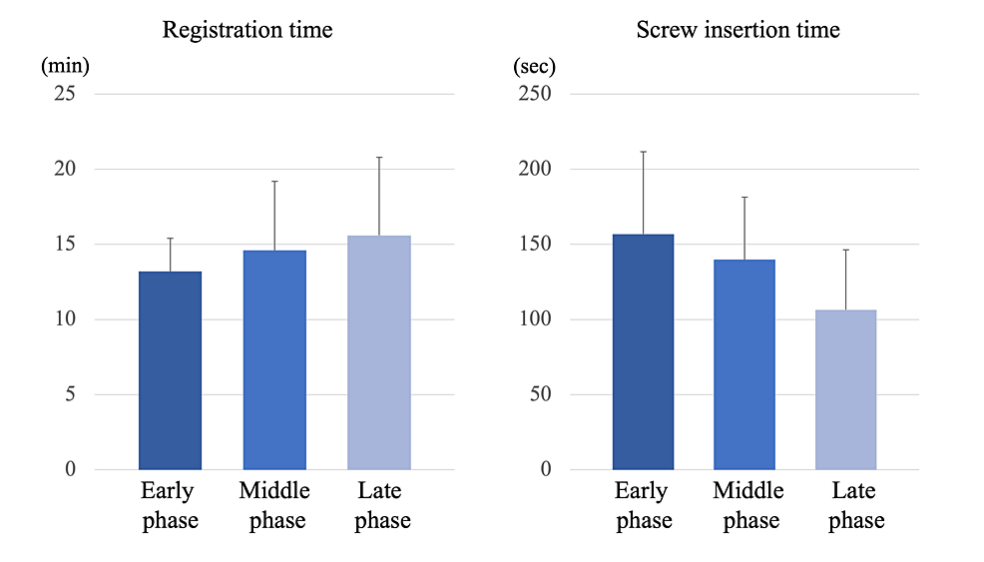
Registration time and screw insertion time There was no significant difference in the registration time among the three groups. There was no significant difference in the screw insertion time among the three groups, but it decreased with experience. The screw insertion time of the late phase tended to be shorter than that of the early phase (p=0.052).

There was no significant difference in the registration time (early phase: 13.2 ± 2.2 min, middle phase: 14.6 ± 4.6 min, late phase: 15.6 ± 5.2 min, p=0.451) among the three groups. There was no significant difference in the screw insertion time among the three groups, but it decreased with experience (early phase: 156.9 ± 54.7 sec, middle phase: 139.9 ± 41.6 sec, late phase: 106.4 ± 39.9 sec, p=0.060) (Figure 6). The screw insertion time of the late phase tended to be shorter than that of the early phase (p=0.052). 

## Discussion

Robotic-assisted CBT screw placement with one- or two-level PLIF had a deviation rate of 1.4%. The deviation rate was 3.8% in the early phase, 0% in the middle phase, and 0% in the late phase. Although there was no difference in operative time or intraoperative blood loss, the screw insertion time in the early 10 cases tended to be longer than that in the late 10 cases. Therefore, it was thought that robotic-assisted CBT screw placement would be proficient in the initial stages after passing the experience of 10 cases.

The deviation rates of CBT screw placement with the freehand technique have been reported to range from 3.3% to 22% [1,18,19]. Dayani et al. reported a deviation rate of 7% in 22 patients using the C-arm, with a tendency for more deviations and complications in the early 11 cases compared with the late 11 cases [20]. Since CBT is different from conventional PS trajectory, computer-assisted devices such as navigation and 3D-printed templates are being used. The deviation rates of the CBT screw placement were reported to be 1.7%-6.7% for navigation [5,14] and 4% for 3D-printed templates [10]. Kumar et al. reported that the CBT screw placement with intraoperative CT navigation significantly reduced screw-related complications compared with fluoroscopy guidance [7]. These computer-assisted devices have helped improve the accuracy of CBT screws and reduce complications from misplacement. 

 In recent years, robotic-assisted CBT screw placement has been reported. Li et al. reported that the accuracy of the CBT screw placement was higher in robotics than that in fluoroscopy [16]. Le et al. found that 87.2% of the CBT screws were in the perfect position (Grade A) with robotics, and the percentage of clinically acceptable screws (Grade A or B) was higher in robotics than that in fluoroscopy [12]. Khan et al. reported that robotics and navigation were equally accurate [14]. These studies reported the accuracy of robotic-assisted CBT screw placement but did not report its learning curve. In this study, the screw insertion time in the early 10 cases tended to be longer than that in the late 10 cases, but the deviation rate was low even in the early period. Therefore, this study concluded that robotic-assisted CBT screw placement was proficient in the initial stages after passing the experience of 10 cases.

The learning curve for robotic-assisted traditional PS placement has been reported to require 0 to 30 cases of experience [21,22]. In this study, robotic-assisted CBT screw placement would be proficient in the initial stages after passing the experience of 10 cases, which may mean that the number of cases required for the learning curve is less than robot-assisted traditional PS placement. We performed robotic-assisted CBT screw placement after 30 cases of robotic-assisted traditional PS placement for a variety of conditions, including fractures, spinal deformities, and degenerative diseases. The experience with robotic-assisted traditional PS placement might influence proficiency in robotic-assisted CBT screw placement.

This study had several limitations. This study was a retrospective study, not an early-stage prospective study of robotic-assisted spine surgery. The number of cases in this study was small. The G*Power 3.1 software was applied to power analysis. The significance level α was set at 0.05, the power (1-β) was set at 0.8, and the effect size was set at 0.4. The total sample size required for the analysis of variance in the three groups was 66. We did not perform an assessment of bone mineral density. We could not compare with conventional CBT screw placement with C-arm images because we have no data of conventional CBT screw placement including the insertion time or fluoroscopic time. The screw insertion was performed by seven surgeons. There may have been differences in screw insertion time and accuracy among surgeons. In the future, we plan to look into how different surgeons' techniques affect how quickly and accurately screws are inserted. We did not evaluate the screw length to confirm whether an ideal CBT trajectory was obtained. The screw length is associated with biomechanics stability and may be relevant to the clinical outcome. We did not evaluate the relationship between screw accuracy and clinical outcome. In the future, we plan to investigate the clinical outcome of PLIF with robotic-assisted CBT screw placement. 

## Conclusions

The deviation rate of robotic-assisted CBT screw placement with one- or two-level PLIF was 1.4%, which was highly accurate. The deviation rate was 3.8% in the early phase, 0% in the middle phase, and 0% in the late phase. Although the deviation rate was low even in the early period, the screw insertion time in the early 10 cases tended to be longer than that in the late 10 cases. After passing the experience of 10 cases, this study concluded that robotic-assisted CBT screw placement was proficient.
